# Who really decides? Feeding decisions ‘made’ by caregivers of children with cerebral palsy

**DOI:** 10.4102/sajcd.v71i1.1001

**Published:** 2024-03-18

**Authors:** Lavanya Naidoo, Mershen Pillay, Urisha Naidoo

**Affiliations:** 1 Discipline of Speech-Language Therapy, School of Health Sciences, University of KwaZulu-Natal, Durban, South Africa; 2 Department of Speech Language Pathology, Faculty of Humanities, University of the Witwatersrand, Johannesburg, South Africa; 3 Department of Speech and Language Therapy, Institute of Education, Massey University, Auckland, Palmerston, New Zealand

**Keywords:** children, enteral feeding, caregivers, decision-making, South Africa

## Abstract

**Background:**

There are no definitive guidelines for clinical decisions for children with cerebral palsy (CP) requiring enteral feeds. Traditionally, medical doctors made enteral feeding decisions, while patients were essentially treated passively within a paternalistic ‘doctor knows best’ approach. Although a more collaborative approach to decision-making has been promoted globally as the favoured model among healthcare professionals, little is known about how these decisions are currently made practically.

**Objectives:**

This study aimed to identify the significant individuals, factors and views involved in the enteral feeding decision-making process for caregivers of children with CP within the South African public healthcare sector.

**Method:**

A single-case research design was used in this qualitative explorative study. Data were collected using semi-structured interviews and analysed using reflexive thematic analysis.

**Results:**

Four primary individuals were identified by the caregivers in the decision-making process: doctors, speech therapists, caregivers’ families and God. Four factors were identified as extrinsically motivating: (1) physiological factors, (2) nutritional factors, (3) financial factors and (4) environmental factors. Two views were identified as intrinsically motivating: personal beliefs regarding enteral feeding tubes, and feelings of fear and isolation.

**Conclusion:**

Enteral feeding decision-making within the South African public healthcare sector is currently still dominated by a paternalistic approach, endorsed by a lack of caregiver knowledge, distinct patient-healthcare provider power imbalances and prescriptive multidisciplinary healthcare dialogues.

**Contribution:**

This study has implications for clinical practice, curriculum development at higher education training facilities, and institutional policy changes and development, thereby contributing to the current knowledge and clinical gap(s) in the area.

## Introduction

The following vignette was developed from the first author’s own clinical experience and from reviewing the literature (Appendix 1):

The doctor arrives. He is tall and walks past the patients quickly, with large black circles under his eyes darkening his gaze as he glares at the office door. He sits down, picks up the first file and starts shouting at the nurses.

The queue begins to move slowly.

It is 12:00 and she walks into his office, ‘Hlala panzi [*sit down*]’, he says and gestures to a chair with a slight smile. He reads the file and then motions for her to put the child on the bed.

He examines the child.

[*Silence*]

He goes back to his desk to write down his thoughts.

[*Silence*]

Then he looks at the nurse. ‘Please tell her the child is going to be admitted. He has a serious infection. She must stay with him while he is here. He will probably need a PEG – a food pipe from the stomach’.

The nurse, standing beside him, translates.

She looks at the nurse, bewildered. She has no money, no phone, no clothes. How can she stay? How can she leave him? She begins to cry.

The doctor looks up from his desk, visibly irritated by her ‘emotional outburst’. ‘He [*pointing at her son*] can’t eat. He is too thin. He needs to get food. The pipe will help make him better to go home soon’.

[*Silence*]

‘Do you understand? Do you agree to this?’

She looks down, tears still streaming down her face. ‘Yes’.

The above mentioned vignette serves to illustrate how feeding decisions within the South African public healthcare sector are intimately contextualised to how people’s relationships have developed historically as linked to the impact of apartheid in the country. It is a history dominated by inequities and entrenched in legislation, the remnants of which remain. South Africa’s current healthcare system reflects these inequities. Clinical decision-making is understood as the process whereby healthcare professionals use their clinical and biomedical knowledge, consider risk-benefits, probabilities and various outcomes to problem-solve within dynamic contexts to make choices and identify an evidence-based course of action (Tiffen et al., 2014). The public healthcare sector, for example, services the majority of the population (84%) and yet only employs 22% of the country’s speech-language therapists (SLTs) nationally, the majority of whom are white (59.7%) (Pillay et al., 2020). Furthermore, the public healthcare sector depends entirely upon the state for funding, whereas the private healthcare sector is financed privately and services a minority of the population (16%) (White Paper on National Health Insurance, 2015). Additionally, traditional healers provide services to a significant portion of the population in South Africa (72%) (Mander et al., 2007) with little government-allocated infrastructure or funding (*Traditional Health Practitioners Act* [Republic of South Africa, 2008]).

This pattern of healthcare access perpetuates racial discrimination in a country where the majority of poor black African people are reliant on public healthcare services (Mhlanga & Garidzirai, 2020). This service access imbalance is reminiscent of the healthcare system during the apartheid era (1948–1993), when colonial subjugation, apartheid health policies and apartheid dispossession polarised the healthcare sector (Coovadia et al., 2009). Service provision during that period was fragmented, racially biased and sub-standard (Maphumulo & Bhengu, 2019), with a distinctive power imbalance underscoring the healthcare interactions.

Appavoo (2021) argued that the power differential between doctor and patient evolved from unidirectional knowledge, steering patients into a predetermined decision based on the information they received. In this way, healthcare professionals could use their expertise and knowledge to conceal their paternalism. Archard (1990, p. 36) defined paternalism as ‘the usurpation of one person’s choice of their own good by another person’. While South Africa has undeniably made significant strides to improve healthcare provision in recent years (Maphumulo & Bhengu, 2019), the clear argument is that the impact of past racial and socio-political policies and current socio-economic challenges within the country continue to support the power imbalance between healthcare professionals and patients. Additionally, the impact of culture when considering feeding decisions cannot be discredited given the long-standing beliefs and/or practices regarding food and eating that can influence the decision-making process (Kruger & Gerike, 2002).

Healthcare inequities are not exclusive to the apartheid era but have continued into post-apartheid South Africa, despite the policy-related interventions that have since been implemented (Omotoso & Koch, 2018). As was poignantly argued by Maldonado-Torres (2007), coloniality has long survived colonialism through the preservation of power patterns that continue to affect modern practices, as is evident when reviewing the current models to healthcare.

Traditionally, clinical decisions were made in deference to a biomedical model (Pillay & Pillay, 2021) that adhered to a paternalistic, ‘doctor knows best’ approach (Manyonga et al., 2014). This approach has since evolved into a more consumerist model, emphasising patient empowerment, autonomy and the protection of patients’ and consumers’ rights (Rowe & Moodley, 2013). However, a particular criticism of this model is that previously oppressed, disempowered state patients receiving free healthcare services may find it difficult to view themselves as ‘consumers’ and have agency in their healthcare decisions (Rowe & Moodley, 2013).

This study thus aimed to explore the feeding decisions made by at-risk and vulnerable population groups within the South African public healthcare sector, specifically caregivers of children diagnosed with cerebral palsy (CP). The prevalence of children diagnosed with CP in KwaZulu-Natal, South Africa, is estimated at 10:1000 live births under 10 years of age (Couper, 2002). This is believed to be the highest in the world, with the prevalence in African countries ranging from 2:1000–10:1000 live births (Donald et al., 2014), Western countries ranging from 1.5:1000–4:1000 live births, and the overall prevalence globally being approximately 2:1000 live births (Katangwe et al., 2020; Oskoui et al., 2013). Of these children, a majority will have some form of feeding (53.5%) or swallowing (50.4%) difficulty (Speyer et al., 2019), with the prevalence of dysphagia estimated between 25% and 45% in typically developing children, up to 80% in children diagnosed with developmental delays (Cohen & Murray, 2016), and up to 85% of children with a severe neurological disability (Hulst et al., 2022). When considering the prevalence here and given that malnutrition is considerably higher among children with CP in low and middle-income countries (Jahan et al., 2022), the imminent need to cultivate research that is contextually appropriate and relevant to caregivers and healthcare professionals proves significant.

Children with CP have a greater aspiration and malnutrition risk (40% – 50%) (Simpamba, 2017) owing to their feeding and swallowing difficulties. Aspiration, if left untreated, may develop into aspiration pneumonia and, in some cases, can be fatal. Children with CP are categorised as malnourished when they are unable to feed safely for a prescribed period or present with high nutrient needs (Marpole et al., 2020). Enteral feeds are typically recommended using either a nasogastric tube (NGT) or a percutaneous endoscopic gastrostomy (PEG) feeding tube (Welbank & Kurien, 2021). Enteral feeding options are known to improve swallowing safety, nutrition and growth, and to reduce feeding times (Suh et al., 2020), but conversely PEG feeding, for example, has also been linked to increased reflux and aspiration concerns (Nishiwaki et al., 2009), and even significant weight gain secondary to poor management of the feeding apparatus (Lynch & Fang, 2004). Time and cost implications for caregivers are also reported (Taylor et al., 2022), and thereby linked to issues of third-party disability. While these risks point to a need for timeous and collaborative clinical decision-making for these children, an in-depth search revealed that no literature or prescribed guidelines are currently available in South Africa regarding clinical decision-making for children with CP who require PEG feeds.

Most available guidelines in South Africa for enteral feeding decisions focus on adults or neonates (less than 4 weeks old). Guidelines for adults include the South African Society for Parenteral and Enteral Nutrition (SASPEN) statement on nutritional management of patients with severe acute respiratory syndrome coronavirus 2 (SARS-CoV-2) infection (SASPEN, 2020), and the National Department of Health’s Enteral Nutrition Practice: Guidelines for Adults (2016). For neonates, decision-making guidelines are available within the neonatal intensive care: pre-discharge and post-discharge report (Van der Westhuizen et al., 2018). The Miller and Kenny decision-making framework for the involvement of children in health-related research (Kelly & MacKay-Lyons, 2010) and the clinical and ethical four-quadrant approach decision-making model by Jonsen, Sieglar and Winslade in 1982 (Sokol, 2008) offer some guidelines regarding the decision-making processes for children although they are largely Euro-American in their origin. Therefore, the need for current evidence-based literature is critical, particularly when considering paediatric feeding decisions in South Africa.

## Aims and objectives

The study explored how PEG feeding decisions are made by caregivers of children with CP who have feeding and swallowing difficulties. The following objectives operationalised the study’s aim:

- Identification of significant individuals, for caregivers, involved in the feeding decision-making process and their respective roles,
- Identification of caregiver factors (extrinsic motivators) affecting the feeding decision-making process, and the
- Identification of caregiver views (intrinsic motivators) regarding the feeding decision-making process.

## Method

### Research design

A single-case research study design was used to allow for a detailed, holistic, empirically rich and nuanced understanding of this specific phenomenon (feeding decision-making) (Gustafsson, 2017).

### Setting

Participants were recruited from one public healthcare hospital in Pietermaritzburg, KwaZulu-Natal. The facility services the majority of public health patients accessing tertiary services in Northern KwaZulu-Natal for a variety of medical conditions, including paediatric PEG surgeries. Patients at the facility are managed within a collaborative multidisciplinary team approach.

### Study population and sampling strategy

Caregivers of children with CP who required PEG placements secondary to their feeding difficulties were selected as the target population group for this study. Two caregivers were interviewed as part of the pilot study, and two were interviewed during the main study. While the researchers initially hoped to interview eight caregivers (three for the pilot study and five for the main study), various logistical and accessibility challenges drastically scaled down the number of eligible caregivers available for interviews.

Eligible caregivers needed to have met one of the following inclusion criteria: (1) had a child with CP who required a PEG and for whom the PEG had been refused, (2) had a child with CP who required a PEG and for whom a PEG had already been placed, or (3) had a child with CP who required a PEG and had since had their PEG removed. During the participant screening process, it was evident that a number of children with CP seen at the hospital have since had the PEGs removed. As such, it proved necessary to revise the inclusion criteria to include these caregivers also. Additionally, the caregivers needed to be actively involved in their child’s management, have a child 2–6 years of age at the time of the PEG decision, and be proficient in either English or isiZulu. In KwaZulu-Natal, English and isiZulu are the two most widely spoken official languages (KZN Provincial Language Policy, 2008). Children aged between 2 and 6 years of age were specifically selected given that developmentally children from 2 years of age are expected to be able to feed (using utensils) independently, without any assistance, and children with CP are often diagnosed up to 2 years of age (Te Velde et al., 2022). Additionally, Reilly et al. (1996) argued, in their study, that children with CP aged between 1 and 6 years present with significant oral-motor dysfunction, are often entirely dependent on their caregivers for their feeding needs, and present with severe gross motor involvement.

### Pilot study

The novelty of the data collection tool called for a pilot study (Van Teijlingen & Hundley, 2001), the results of which successfully highlighted the aim and objectives of the study and the viability of the research tool. The results of the pilot study drew attention to the need for changes to the interview schedule, including amendments to the study’s logistics and interpreter training. Prior to the initiation of the study, interpreters received training in concept translation as opposed to word-for-word translation. Concept translation entails the translation of information from English to isiZulu that closely mirrors the meaning of the original utterance, in an effort to ensure the reliability of the data collected (Van Nes et al., 2010).

### Data collection

Semi-structured in-person interviews were used as the data collection tool in this study, and were conducted in either English or isiZulu, according to each participant’s preference. An isiZulu interpreter was available throughout to assist participants as needed. An interview schedule was developed to help structure the interviews and was based on the research objectives. Critical incident vignettes were also developed to help facilitate the interviews, but not to dictate a dialogue to the participants. The critical incident vignettes were developed around a case discussion of a child with CP who required a PEG placement.

Participant responses were audio-recorded throughout the interviews, which were approximately 90 min in length. The interviews were scheduled during the participants’ follow-up consultations at the hospital. Throughout the study, the caregivers were reminded of the voluntary nature of their participation in the study and how to withdraw from the study at any point should they so wish.

Participant interviews were envisioned on the same day as the caregiver’s follow-up appointments at the hospital. Referrals for additional psychosocial support interventions could be made as part of the established system at the hospital. Participants who demonstrated any emotional distress and/or who indicated a desire for additional support here could immediately be referred to an onsite staff member for assistance.

### Data analysis

The data were analysed according to the reflexive thematic analysis phases as argued by Braun and Clarke (2006). All digital data were transcribed verbatim to enable familiarisation with the data. All identifying details were anonymised. The transcribed data were then labelled and organised to generate initial codes. The emerging codes were then sorted such that meaningful themes could be identified. The emerging themes were then reviewed and compared to identify overlapping ideas and arguments, and were re-categorised where necessary (Braun & Clarke, 2019). The identified themes were then defined, named and reported as consistent with the research question. Frequent member checks among all researchers was conducted to ensure objectivity and consensus regarding the emergent themes.

### Trustworthiness

The trustworthiness of the research was upheld by ensuring the credibility of the results. Credibility was maintained throughout with: (1) a reflective and iterative line of questioning throughout the interviews; (2) a prolonged engagement using a pilot study which enabled a rapport with hospital staff, and a familiarity with the operations and culture of the hospital; (3) participant honesty measures (iterative dialogues) throughout the interviews; (4) communicating the independent status of the researcher and interpreter to the participants prior to the onset of the study, thereby encouraging them to share their experiences and views without anxiety or fear of losing credibility with any hospital personnel; (5) frequent researcher–supervisor debriefing sessions; (6) peer scrutiny between the researchers to broaden the aims and perspective envisioned for the study; (7) reflective commentary; and (8) member checks (Shenton, 2004) discussing alternative approaches to challenges experienced, highlighting prospective flaws in the study, assisting with developing ideas and preventing researcher preferences or bias.

Dependability was maintained by detailing all aspects of the methodology and data analysis, thereby ensuring the transferability of the study results if duplicated (Shenton, 2004). Investigator triangulation, among all researchers, was frequently and proactively sought throughout the data analysis process to ensure the trustworthiness of the data (Miles et al., 2014). To reduce the risk of bias, conformability was established by encouraging participant reflexive commentary. Researcher reflexivity was also valued, and iterative interviews were frequently utilised to ensure the credibility of the research design and strengthen arguments made throughout. Peer scrutiny by colleagues, academics and peers was also encouraged, as it allowed for the opportunity to challenge assumptions (Shenton, 2004).

### Ethical considerations

Ethical approval was granted by the University of KwaZulu-Natal Biomedical Research Ethics Committee (BE424/15). Gatekeeper permission was obtained from the hospital management and the Provincial Department of Health; written consent for both interviews and audio-recordings was required prior to the initiation of the data collection process.

## Results and discussion

The results are presented based on the study’s three objectives in relation to significant individuals and their respective roles, caregiver factors and caregiver views on the clinical decision-making process. The responses from the two caregivers are discussed within each section.

## Significant individuals and their roles

**FIGURE 1:**
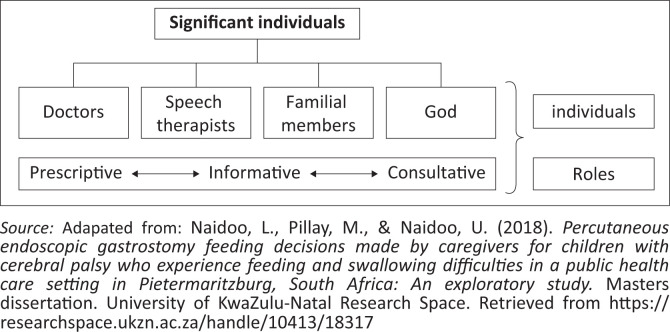
Representation of significant individuals and their roles.

During the interviews, four groups of significant individuals were identified: doctors, speech therapists, family members and God, and their respective roles were discussed (Figure 1).

### Doctors

#### ‘They [*doctors*] all told me’

The doctors used medical jargon:

‘They [*doctors*] all told me the PEG [*percutaneous endoscopic gastrostomy*] would help him with this and this and this.’ (P1, primary caregiver, female)

Prescriptive dialogues – ‘told me’ – were used as techniques to enforce their decisions onto their patients, thus positioning themselves in a dominant role. This distinct power imbalance was a recurring theme for both caregivers interviewed.

In this study, the caregiver was the one being ‘told’ about the decisions made. Therefore, she understood her role submissively and in contrast with the doctor, who had a more knowledgeable and authoritative role. The nature of this interaction between the doctor and caregiver is premised on the unidirectional flow of knowledge from the expert to the passive, sick patient and their family. The caregiver’s use of the phrase ‘this and this and this’ during the interview was discursively analysed and demonstrated that while she did not understand the medical terms used, she was still amenable to the decisions made. Patients often defer to doctors’ decisions made on their behalf owing to the doctors’ medical expertise and may view their own knowledge as irrelevant to the decision-making process (Lian et al., 2022). The comprehension and communication of medical jargon is a well-documented barrier for caregivers, particularly non-English speaking caregivers of children with special needs (Sezgin et al., 2020). Medical jargon is often obscure for caregivers and can cause them undue trauma and distress (Tong et al., 2020). These results provide an example of the extent to which paternalism and the power dynamics that sustain it continue to permeate the public healthcare sector in South Africa.

The results also indicated that caregivers perceived themselves as the perpetrators of their children’s difficulties:

‘When I give him food, he was getting so thin.’ (P1, primary caregiver, female)

As opposed to the doctors who are viewed as capable of preserving life:

‘When they put all the things, yes he [*the child*] was alright.’ (P1, primary caregiver, female)

This resonates strongly with the notion of strategic essentialism, which details the way in which agency is created among individuals (Spivak, 1988). In this study, strategic essentialism was revealed in the way caregivers sought to cement their submissive role, thus compounding the power dynamic between them and their doctors:

‘… I wish that I lived here and didn’t live so far away, because they treated us like their own children … when I have questions, I can ask here. When we go home, it’s not the same.’ (P1, primary caregiver, female)

Caregivers of children with CP, who are empowered to view themselves as proactive participants within the decision-making process have greater understanding and realistic expectations about their child’s medical diagnosis and quality of life post-hospitalisation (Wittenberg et al., 2020). However, not all caregivers share this opinion. In a study by Beecham (2000), the caregiver viewed her child’s healthcare professionals as the perpetrators of the child’s difficulties and held them responsible, considering herself independent of the doctors.

The results clearly reveal that the medical jargon used by the healthcare professionals in this example established their positionality within the clinical interaction, and thus their social power and paternalism. Thus, healthcare professionals should be cognisant of this intersectionality to better acknowledge and manage differences, and help caregivers in making more well-informed and sustainable decisions.

### Speech therapists

#### ‘They taught us’

The speech therapists’ role was understood by the caregivers as informative and supportive of the decision already taken by the doctors:

‘They taught us, before they put the PEG in, about what is going to happen.’ (P1, primary caregiver, female)

While the benefits of gastrostomy feeds have been widely documented, they cannot be positioned as the only alternative to ensure nutritional gains. Healthcare professionals are obligated to inform their patients impartially about all aspects of their care and to support them throughout the decision-making process (The National Patient’s Rights Charter, 1999). The inference here is that when speech therapists use their medical knowledge to endorse a pre-emptive decision, they inadvertently reinforce the power dynamic between themselves and the caregivers. Within this clinical interaction, the speech therapists are positioned as medical experts, and the caregivers as the recipients of the suggested treatment option, implying a unilateral transfer of medical decisions from the medical doctors to the speech therapists to the caregivers. The caregivers were thus more likely to comply with the decisions made when endorsed by both expert parties – the speech therapists and medical doctors – despite the clear power imbalance.

### Family members

#### ‘They all had to make the decisions together’

Caregivers and their children form part of an extended family unit, which is frequently consulted when making decisions (Ketani & Tanga, 2018). When asked about who ultimately decided on the PEG placement for her child, one caregiver responded with:

‘… the grandmother.’ (P2, primary caregiver, female)

This is not uncommon and is also practised elsewhere in the world among Hispanic (Kagawa-Singer & Blackhall, 2001), African American (Orlovic et al., 2019) and Korean American (Blackhall et al., 1995) families. According to Statistics South Africa (2018), 55.3% of black African children (aged 0–6 years) living in South Africa currently reside in extended family units (single parents or spousal couples living with their own children and other family members). This statistic is not surprising given the African philosophy of ubuntu – the idea that ‘a person is a person through other persons’. Ubuntu illustrates how the connectedness between black African communities and families is established and valued, while cultivating a sense of individuality from childhood (Mugumbate & Chereni, 2019).

Extended family members in black African families frequently include grandparents, uncles, aunts, cousins and in-laws, as well as people not related to the family but who reside in the same region (Mafumbate, 2019). Even within these familial structures, caregivers are often marginalised in deference to cultural or socioeconomic practices (Jama et al., 2018). While the study aims to comment on feeding decision-making practices within the public healthcare sector, it is evident that even within the home environment, caregivers can be subdued into a submissive role:

‘The [*child’s*] grandmother also lives with them, and they all had to make the decisions together.’ (P2, primary caregiver, female)

In this way, the inherent power imbalance experienced by caregivers is sustained within the family unit, albeit to a lesser extent, although undeniably still noticeable.

### God

#### ‘… the plan of God’

God was viewed as a pervading, omniscient factor throughout the decision-making process. The caregivers in the study often referred to God as having an authoritative role:

‘I kept telling myself it was all the plan of God.’ (P1, primary caregiver, female)

Different cultures and religions often have long-held beliefs, practices and behaviours regarding food and eating that can influence the decision-making process (Kruger & Gericke, 2002). The impact of religion on clinical decision-making has been well documented with some religions known to comment on, for example, decisions made regarding medicines used, the gender of preferred healthcare professionals and the patient’s diet (Geros-Willfond et al., 2017; Rego et al., 2020; Sheldrick, 2007; Swihart et al., 2022). When considering the impact of religion and spirituality on clinical decision-making, caregivers noticeably deferred to a higher being (God) as a coping mechanism to help sustain them as they care for their child:

‘Even when something is wrong, I must smile and I always smile … I just pray to God to keep us and keep us carrying on.’ (P1, primary caregiver, female)

Masuku and Khoza-Shangase (2018, p. 247) argued that caregivers (when caring for a family member with a disability) believe the challenges they are experiencing are ‘from God’ and a ‘test of their faith and sense of humanity’. Religion is thus viewed as a support system to enable resilience among caregivers (Karaca & Şener, 2019). God and spirituality have authority and value regarding clinical decision-making and reflect how religion has positioned itself in a medical context.

## Caregiver factors

**FIGURE 2:**
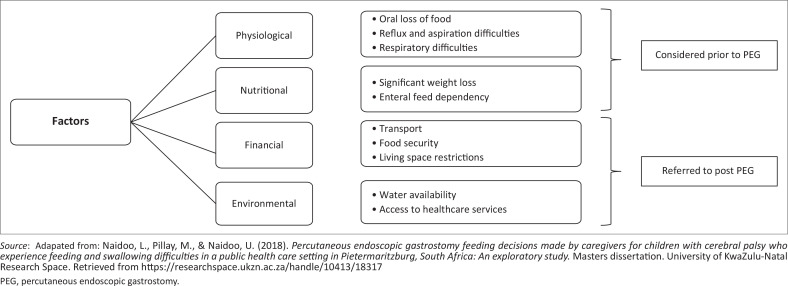
Hierarchical depiction of caregiver factors.

Four categories identified under the caregiver factors were: physiological, nutritional, financial and environmental factors, as presented in Figure 2.

### Physiological factors

#### ‘… he can’t eat with the mouth’

Physical symptoms during feeds, such as the loss of food orally, coughing or vomiting, carry significance for caregivers. The results from the study indicate that for one caregiver, the PEG placement was necessitated by the child’s inability to feed orally:

‘He can’t eat with the mouth. Every time he eats with the mouth, everything comes out. This is why they put a PEG.’ (P1, primary caregiver, female)

Caregivers were often concerned about the stigma that their children experience:

‘Sometimes our children are not recognised by people because people are scared of our children.’ (P1, primary caregiver, female)

This concern was supported frequently in the literature (Dadich et al., 2022) where caregivers of children with disabilities reported concern that their child may be regarded as ‘less human’ post-PEG placement (Petersen et al., 2006, p. 716). They also perceived oral feeding as a means to enjoy eating, without which overall familial bonding, positive meal experiences and social interactions may diminish (Shune & Namasivayam-MacDonald, 2020). Eating is understood as gratifying, and oral feeding is considered a positive and pleasurable activity. But why is this relevant? Because it echoes caregivers’ concerns that their child(ren) will be viewed ‘differently’ and ‘less than’ when compared to others. Therefore, it is valuable to explore physical symptoms, coupled with caregiver beliefs, when considering clinical decision-making, given how they influence caregivers and inadvertently endorse a distinct power imbalance.

#### ‘… was not breathing well’

Respiratory concerns or symptoms were also reported. One caregiver alluded to feelings of fear, pain and ridicule from others when her child presented with respiratory difficulties:

‘Even when someone was walking past the room, they would come back when they heard his breathing; it was so bad … It was painful.’ (P1, primary caregiver, female)

In their study, Tong et al. (2020) linked this to the theme of provoking and exacerbating undue trauma in patients or caregivers. Respiratory complications are frequently reported as a contributing malnutrition concern in children with CP (Boel et al., 2018), and are arguably confusing, stressful and distressing for caregivers. Tong et al. (2020) posited that trauma is often provoked or exacerbated in patients when motivated by various factors. These included fear of the unknown, impending death, despair, premature assumption and labelling, and stigma, judegement, and failure of self:

‘… it was so bad … [*the child*] was not breathing well … It was painful because I was like a video or TV.’ (P1, primary caregiver, female)

The child’s respiration difficulties speak to the vulnerability of caregivers in a clinical context, where they are tasked with making key decisions for their children while navigating a myriad of other factors. It is crucial, therefore, that such factors are adequately considered throughout the decision-making process.

### Nutritional factors

#### ‘… he was getting so thin’

Weight was identified as a common theme throughout the decision-making process, and was alluded to by both caregivers:

‘There was no development or weight gain … He weighed almost 9 kg at 3 years old before the PEG.’ (P2, primary caregiver, female)

Traditionally, people viewed underweight children with concern, while associating overweight children with notions of power and wealth (Klingberg et al., 2020). A study by Ramgolam (2018) on obesity and body image perceptions among black South African nurses in KwaZulu-Natal found that overweight people were assumed to be strong, healthy, and wealthy. However, being underweight was symbolic of poverty and unhappiness, and possibly indicative of someone with human immunodeficiency virus (HIV) or acquired immunodeficiency syndrome (AIDS). These outdated stereotypes have since been disputed, with social media campaigns and contemporary Western influences supporting leaner body sizes among South African youth and favouring healthier lifestyles (Cohen et al., 2020).

Despite these shifting viewpoints, the data suggests that, for children diagnosed with CP, weight loss remained a significant indicator of poor health and one that required urgent intervention. For many healthcare professionals, weight is arguably a quantitative statistic that objectively guides their decision-making process. However, as illustrated, it has symbolic, traditional and emotional significance for many caregivers, and should not be viewed in subjective isolation.

### Financial factors

#### ‘I don’t have money’

Financial considerations were an inescapable reality for caregivers when considering feeding decisions. They referred primarily to transport difficulties, food security and living space restrictions.

The one participant stated that:

‘I also have to save to come here.’ (P1, primary caregiver, female)

The inaccessibility of public health services for children with disabilities in South Africa is well documented (Coutts & Solomon, 2020; Vergunst et al., 2015), and linked to the discrimination that many caregivers and children experience when trying to use public transport in rural areas. Often, the only alternative for caregivers is to use expensive private transport options to access basic services:

‘I can’t come with the taxi because they won’t take us from the road.’ (P1, primary caregiver, female)

This often proves to be a costly endeavour for caregivers who are dependent on social grants for their children:

‘I hire the taxi car for R200 to go. Even to attend the CP clinic, I must hire that car with that money.’ (P1, primary caregiver, female)

Consequently, many caregivers forego necessary follow-up consultations for their children owing to a lack of funds. While this point did not link directly to the PEG placement, it is significant given the practical challenges caregivers experience, which undoubtedly affects their decision-making process.

#### ‘… not eating every kind of food’

The caregivers spoke of food security with reference to procuring adequate, affordable and nutritious food. One caregiver was acutely aware that her child was unable to eat certain foods, and that she was required to attend her local hospital to access food sources:

‘[*The child*] is not eating every kind of food. Some food I take from the provincial hospital.’ (P2, primary caregiver, female)

It is interesting to note how the hospital has thus created a level of dependency on the caregiver. For each hospital visit, the caregiver is required to spend approximately 8% of her total monthly income. Additionally, this is contingent on the availability of the food at the hospital, failing which the caregiver would need to source the food privately and at an additional cost.

One participant stated:

‘We are full at home.’ (P1, primary caregiver, female)

Living space is equated with an improved quality of life. One caregiver attributed her lack of financial resources to her inability to provide her child with the room she believed he required for his care:

‘I wish I had a space for [*the child*] … but how can I because I don’t have money.’ (P1, primary caregiver, female)

A study conducted by Ismail et al. (2022) found that the estimated cost per year, in Malaysia, for a child with CP is more than RM 52 549 (approximately R189 367). While no information regarding costs specific to South Africa is currently available, it may be equated with Malaysia, given that both are categorised as middle-income countries. Raphulu et al. (2021) pointed out that unemployed caregivers are reliant on their child’s dependency grant (for children with CP) of R1890 and the child support grant of R460 per month. Unsurprisingly, they found it financially challenging to care for all the child’s needs and transport between medical appointments.

The reason this is explained in such detail with no reference to PEG placements is that caregivers do not exist in a vacuum. This study’s results speak to how caregivers often have to consider a multitude of financial factors over and above their child’s feeding and swallowing difficulties. The data source lends itself to the influence of real-world experiences on caregivers, something that healthcare professionals need to consider when making decisions for their patients. Without adequate psycho-social support and education, caregivers can often feel depressed, isolated and powerless, compounding their dependency on their doctors and the public healthcare system.

### Environmental factors

#### ‘… any kind of water’

Access to clean water was also referred to in conversation with the caregivers when considering childcare and general hygiene. Regrettably, access to services is often a challenge for those living in rural areas, and exponentially greater for those with disabilities (Vergunst et al., 2015).

A participant stated that:

‘… they have to take water from the [*rivers*], any kind of water.’ (P1, primary caregiver, female)

This caregiver identified access to water as vital to the care of her child. The obvious inference is that a lack of access may influence the caregiver’s ability to decide in favour of the PEG feeding tube because she believed her environmental restrictions may limit her child’s ability to benefit from this intervention. While this comment did not link directly to the PEG decision-making process, it cannot be ignored as it speaks to the caregiver’s mindset and practical considerations that may affect her decision.

#### ‘I wish that I lived here and didn’t live so far away’

Access to healthcare services was highlighted as an additional environmental challenge. One caregiver referred to the fact that she and her child lived far away from the hospital, even elaborating that the level of care they received at this hospital differed from the care they received at their local hospital:

‘Even when I have questions, I can ask here. When we go home, it’s not the same.’ (P1, primary caregiver, female)

While this point links strongly to the transport theme mentioned earlier, it differs in that this statement was not made regarding distance or financial constraints, but rather in support of accessing credible services because the caregiver believed she would receive better care. With reference to the PEG decision, while not explicitly linked, the plausible assumption was that the caregiver possibly felt as though the PEG feeding tube would require additional medical monitoring, which was inaccessible to her and her child at home. It is noteworthy that the doctors at the preferred hospital had cultivated a level of dependency from the caregiver by assuming a parental role:

‘… they they treated us like their own children.’ *(P1, primary caregiver, female)*

This type of parental-child relationship suggests protection and authority, compounding the caregiver’s dependence on the doctor.

## Caregiver views

**FIGURE 3:**
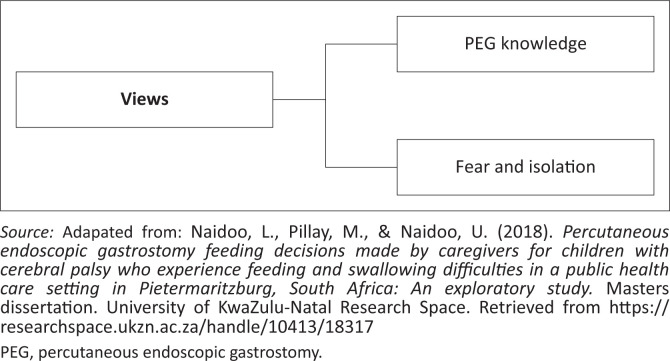
Representation of the caregivers’ views.

Two emergent themes were identified, as represented in Figure 3.

### Percutaneous endoscopic gastrostomy knowledge

#### ‘It was new’

Caregivers lack basic knowledge about their child’s diagnosis, often resulting in unrealistic expectations about their child’s functioning (Savage et al., 2021). Both interviewed caregivers referred to the PEG as new and unfamiliar, adding that they had not been exposed to a PEG feeding tube before:

‘It was new, and they [*the family*] didn’t know about it.’ (P2, primary caregiver, female)

Shune and Namasivayam-MacDonald (2020) argued that caregivers often experienced anxiety concerning their increased responsibilities, changing roles and feelings of being ill-prepared.

Caregivers often felt they lacked the medical knowledge and skills to reproach their doctor once a decision had been made, highlighting the impact of paternalism on PEG feeding decision-making. This theme intersects with the familial support theme mentioned earlier, where caregivers were at times socially isolated from their families owing to a lack of knowledge regarding the PEG feeding tube (Savage et al., 2021). In such instances, the task of caring for the child fell to the caregiver exclusively. Without adequate knowledge or education, cultural myths were allowed to develop. In a study by Savage et al. (2021), the participants alluded to how their neighbours spiritually attacked them, which they believed resulted in their child having CP. Fleming and Ledogar (2008) argued that in such cases, the practising of indigenous spirituality and traditions could serve as a protective measure to promote community, family and individual resilience.

Percutaneous endoscopic gastrostomy knowledge does not simply link to the need for caregivers to know more about the workings of the PEG feeding tube. It points to a larger conversation of how some caregivers are not being empowered within the confines of their spiritual understanding to acquire knowledge regarding the child’s condition. Healthcare professionals and religious leaders should, therefore, be encouraged to collaborate with caregivers such that decisions made are respectful and inclusive.

Despite the caregivers’ lack of knowledge regarding the PEG, they were still able to demonstrate personal resilience and a commendable ability to negotiate an alien healthcare system:

‘I told myself not to be scared and that all the time I must be happy. Even when something is wrong, I must smile. And I always smile and then the people look at [*the child*] and say, ‘He’s so big, he is so beautiful with so nice clothes’ and then they just forget about the trachy and all this stuff.’ (P1, primary caregiver, female)

The inference here is that despite the PEG being new and unfamiliar to the caregiver, they were still able to navigate through their feelings of uncertainty (and familial uncertainty) to manage their child’s difficulties capably:

‘People are scared of our children sometimes, but when there is education for the mother, then they [*the people*] look at her and say, “She’s a strong one” and I try when something is wrong with [*the child*].’ (P1, primary caregiver, female)

### Fear and isolation

#### ‘No-one at home saw me crying’

Fear and isolation underscore the decision-making process for many caregivers. One caregiver appeared almost obligated to present herself as optimistic for fear that her child would not be accepted or stigmatised:

‘Sometimes our children are not recognised by people because people are scared of our children.’ (P1, primary caregiver, female)

The stigma and fear that many caregivers of children with CP experience within their families and communities has been well documented (Dako-Gyeke et al., 2021; Vadivelan et al., 2020). One caregiver reported concern regarding her child’s welfare if others saw her cry:

‘Even if you see that there is something wrong, you must tell yourself to be strong. For me, when something was wrong, no one at home saw me crying. I always went out of the house to cry and wiped my face before going back into the house. I was worried what would happen to [*the child*] if I cried in the house.’ (P1, primary caregiver, female)

Cankurtaran et al. (2021) argued how the quality of life for mothers of children with CP was often negatively affected because of fatigue, the burden of care, and physiological symptoms:

‘I told myself not to be scared and that all the time I must be happy.’ (P1, primary caregiver, female)

This resonates with the views of Vadivelan et al. (2020), who argued that mothers of children with CP experience personal guilt, worry and blame owing to their child’s disability, and also at a societal level. This finding links strongly with the theory underpinning third-party disability studies for caregivers (Nund et al., 2014).

## Significance and limitations

This research study is unique in that no other study exploring PEG feeding clinical decision-making within the paediatric CP population has been conducted in South Africa previously. While several studies have offered some guidelines for healthcare professionals to use during the PEG decision-making process, they are largely subjective and/or primarily based on Western influences. On the other hand, this study highlights paternalistic realities and champions a practical, collaborative decision-making approach.

While the data is relevant to the study objectives, the lack of variability in the participant sample is acknowledged. Reasons for this lack of participant variation included: (1) participants being recruited from one hospital only; (2) incorrect contact numbers provided by some caregivers who could then not be contacted; (3) lack of follow-up consultations at the hospital; (4) caregiver financial difficulties resulting in poor attendance for follow-up consultations; (5) additional conflicting appointments preventing caregiver attendance during follow-up consultations; and (6) an increase in the childhood mortality rate. However, given the novel nature of the study’s aim, the smaller sample size was deemed appropriate here. As stated by Vasileiou et al. (2018), smaller participant samples were often purposively selected for their ability to elicit phenomenon-specific, richly textured information.

## Implications and recommendations

The study highlights the need for practical initiatives regarding clinical decision-making. In their efforts to manage their patients effectively, healthcare professionals need to be cognisant of the intrinsic views and extrinsic factors that motivate caregivers and heighten their dependency on their doctors, and work towards implementing practical and sustainable strategies to improve caregiver knowledge and empowerment. Table 1 provides examples of such practical initiatives.

**TABLE 1:** Practical suggestions for healthcare professionals to include during caregiver training.

Variable	Suggestion
Significant individuals	Collaborative decision-making Multidisciplinary involvement Familial and religious support structures
Caregiver factors	Education regarding diagnosis and all aspects of intervention Quality of life realities post PEG insertion for both the caregiver and child Financial implications and resources to assist Environmental support systems for the caregiver
Caregiver views	Peer support systems for caregivers, such as the Malamulele Onward peer-supporter programme, which proactively seeks to encourage parental opportunities for the sharing of personal struggles and stories (Saloojee & Bezuidenhout, 2020) Education regarding local healthcare systems for caregivers to access services, such as the Hambisela Project, which specifically centres on caregivers of children with CP in low-resourced communities

*Source:* Adapated from: Naidoo, L., Pillay, M., & Naidoo, U. (2018). *Percutaneous endoscopic gastrostomy feeding decisions made by caregivers for children with cerebral palsy who experience feeding and swallowing difficulties in a public health care setting in Pietermaritzburg, South Africa: An exploratory study.* Masters dissertation. University of KwaZulu-Natal Research Space. Retrieved from https://researchspace.ukzn.ac.za/handle/10413/18317

PEG, percutaneous endoscopic gastrostomy; CP, cerebral palsy.

The results have also highlighted how access to healthcare facilities remains a challenge for caregivers of children with CP living in rural areas. The geographical barriers (terrain, transport and distance) facing rural communities trying to access services remain a perennial challenge (Vergunst et al., 2015). These barriers require urgent attention at a national level, as they can be applied broadly to South African caregivers accessing public healthcare services. The results also emphasise the extent to which practical realities for caregivers are evolving. This points to a continued need, at a tertiary level, for curriculum reform in favour of an Africanised and decolonised model (Moonsamy et al., 2017) to ensure the relevance of knowledge application to the contextual and diverse needs of South African caregivers.

The researchers recommend conducting further research at public and private healthcare facilities within different population groups and using more varied participant samples.

## Conclusion

Power imbalances between doctors and caregivers dominate feeding decisions made within the South African public healthcare sector. Caregivers are often positioned as passive participants for whom decisions are made unilaterally, submissively, and who understand their role as one of passive compliance.

Clinical decisions established on power inequities are not sustainable and risk crippling an already ailing healthcare sector. For healthcare to become relevant, sustainable and user-friendly, healthcare professionals need to consider caregivers and their children as more than beneficiaries for whom decisions are made, and instead as collaborative partners whose input is required and valued, particularly when considering long-term enteral feeds.

## Appendix 1

**TABLE 1-A1:** Supporting references for clinical case exemplar.

Article	Documented support
Stoyanov, J. E. & Edwards, S. D. (2017). Experiences of the health care delivery system in a rural South African Setting. *Journal of Psychology in Africa, 27(4),* 385–387.	Health care providers often report feelings of fatigue characterized by stress, burnout, frustration, anger, hopelessness, anxiety and depression.
Van den Berg, V. L. (2016). Still lost in translation: Language barriers in South African health care remain. *Journal of South African Family Practice, 58(6),* 229–231. Hussey, N. (2012). The language barrier: The overlooked challenge to equitable health care. *South African Health Review 2012/2013, 1,* 198–195.	In multilingual societies, a common barrier to effective communication within the health care sector, arises where healthcare practitioners and their patients do not use the same first language. The authors argue that the patients’ inability to communicate with their healthcare providers effectively adds to their (the patients’) uncertainty, dissatisfaction, and emotional stress.
Ndachi, M. (2014). *Providing health care interpreting in the department of radiation, oncology, Charlotte Maxeke Johannesburg Academic Hospital (unpublished master’s thesis).* University of Witwatersrand. Deumert, A. (2010). ‘It would be nice if they could give us more language’ -– Serving South Africa’s multilingual patient base. *Social Science & Medicine,* 53–61.	Healthcare professionals in public healthcare settings often use *ad hoc* personnel (e.g., nurses) to assist them when a language barrier persists, in an effort to effectively communicate with their patients.
Joseph-Williams N., Elwyn G. & Edwards A. (2014). Knowledge is not power for patients: A systematic review and thematic synthesis of patient-reported barriers and facilitators to shared decision-making, *Patient education counselling, 94(3*), 291-309. Kruger, A., Lemke, S., Phometsi, M., Van’t Riet, H., Pienaar, A. E. & Kotze, G. (2006). Poverty and household food security of Black South African farm workers: The legacy of social inequalities. *Public Health Nutrition, 9(7),* 830–836.	Patients and their families often allowed for this as they perceived their healthcare professionals as being more knowledgeable/authoritative. They were essentially treated passively here and often felt compelled to adopt the role of the ‘good patient’, essentially a submissive and compliant role. Destitute living conditions, financial constraints, and paternalistic structures of the past collectively act to hamper development.
